# Predictors of urinary outcomes following robotic‐assisted laparoscopic prostatectomy

**DOI:** 10.1002/bco2.248

**Published:** 2023-05-06

**Authors:** Dylan Hutchison, Jacqueline Zillioux, Marwan Ali, Jacques Farhi, Anthony DeNovio, David Barquin, David E. Rapp

**Affiliations:** ^1^ Department of Urology University of Virginia Charlottesville Virginia USA; ^2^ School of Medicine University of Virginia Charlottesville Virginia USA

**Keywords:** BMI, nerve sparing, RALP, risk factors, stress incontinence, urge incontinence

## Abstract

**Introduction:**

Incontinence and urgency are common after prostatectomy. The University of Virginia prostatectomy functional outcomes program (PFOP) was developed to comprehensively assess and optimise continence outcomes following robotic‐assisted laparoscopic prostatectomy (RALP). Patients are prospectively evaluated by a Female Pelvic Medicine and Reconstructive Surgery specialist. This study assessed for predictors of 3‐ and 6‐month stress urinary incontinence (SUI) and urgency symptom outcomes following RALP.

**Methods:**

We performed a post hoc review of patients from our PFOP receiving a minimum of 6‐month follow‐up. Urinary symptoms are prospectively assessed using the validated International Consultation on Incontinence Questionnaire‐Male Lower Urinary Tract Symptoms (ICIQ‐MLUTS) questionnaire and daily pad use (pads per day [PPD]). Primary study outcomes included ICIQ‐MLUTS SUI and urgency domain scores and PPD. Multivariable linear regression was performed to identify variables associated with outcomes at 3 and 6 months postoperatively. Variables included patient, oncologic and surgical factors. Each variable was run in a separate model with pelvic floor muscle therapy and surgeon to reduce confounding and prevent overfitting.

**Results:**

Forty men were included. In assessment of ICIQ‐MLUTS SUI domain score, at 3 months, body mass index (BMI) was associated with worse scores, and at 6 months, BMI, hypertension and estimated blood loss (EBL) were associated with worse scores, whereas bilateral nerve‐sparing technique was associated with better scores. For ICIQ‐MLUTS Urgency domain score, at 3 months, preoperative use of benign prostatic hyperplasia (BPH) medication was associated with better scores. No covariates predicted 6‐month ICIQ‐MLUTS Urgency domain scores. For PPD use, at both 3 and 6 months, BMI was a positive predictor, while preoperative use of BPH medication was a negative predictor.

**Conclusion:**

Increased BMI, EBL and hypertension are associated with worsened SUI outcomes following RALP, whereas bilateral nerve‐sparing technique and preoperative BPH medication are associated with improved SUI outcomes. These data may inform patient counselling and help identify patients who may benefit from closer surveillance and earlier anti‐incontinence intervention.

## 1 INTRODUCTION

Incontinence and urgency are common adverse outcomes after prostatectomy and can contribute to patient distress.^1^, ^2^ While many patients have improvement or cure spontaneously with conservative management, many have persistent bothersome symptoms requiring surgical intervention.^3^, ^4^ The American Urologic Association (AUA)/Society of Urodynamics, Female Pelvic Medicine and Urogenital Reconstruction Guideline on Incontinence after Prostate Treatment recommends immediate post‐prostatectomy pelvic floor muscle training (PFMT) and recommends considering surgery as early as 6 months for persistent bothersome symptoms, although many patients are not treated until much later, prolonging distress and reduced quality of life (QOL).^5^, ^6^ The ability to identify baseline and surgical characteristics as predictors of incontinence outcomes will help identify patients who may benefit from closer post‐operative surveillance and timely intervention.

The University of Virginia prostatectomy functional outcomes program (PFOP) was developed to comprehensively assess and optimise continence outcomes following robotic‐assisted laparoscopic prostatectomy (RALP). Patients are evaluated by a Female Pelvic Medicine and Reconstructive Surgery (FPMRS) specialist at baseline, 3, 6 and 12 months following surgery. With this cohort of patients, this study assessed for predictors of 6‐month stress urinary incontinence (SUI) and urgency symptom outcomes following RALP using linear regression.

## 2 METHODS

We performed a retrospective review of patients undergoing RALP for prostate cancer and who were participating in the University of Virginia PFOP.

The University of Virginia PFOP was developed as a clinical initiative to comprehensively assess and optimise functional outcomes following RALP. Accordingly, this programme was created and supervised by a FPMRS specialist (DER). Patients undergoing RALP by any of three surgeons were contacted by the programme team and offered participation in the programme to include in person visits with the FPMRS specialist to allow for comprehensive symptom assessment and in‐person pelvic floor muscle training, dietary counselling, and behavioural training. Those patients declining participation in this fashion were offered virtual follow‐up focused on functional outcomes. In this case, pelvic floor and behavioural education was provided by the patients treating RALP surgeon. Importantly, this programme is unique in that all outcomes were assessed independently by the FPMRS specialist in an effort to optimise accuracy of data and focus on optimising outcomes.

Functional outcomes assessment and surveillance is longitudinally performed in patients undergoing RALP using robust questionnaires, with lower urinary tract symptoms prospectively assessed using the validated International Consultation on Incontinence Questionnaire‐Male Lower Urinary Tract Symptoms (ICIQ‐MLUTS) questionnaire and daily pad use at each follow‐up visit. Questionnaire assessment is performed at baseline and at 3‐week and 3‐, 6‐ and 12‐month timepoints following RALP.

The present study represents a retrospective analysis of data collected as part of this clinical initiative. All patients with 6‐month follow‐up were eligible for analysis. Based on the programme characteristics, the study cohort represents a sample of patients from the overall population of patients undergoing RALP at our institution (40 out of 196 patients undergoing RALP during the analysis period). Multivariate analysis was performed to assess for predictors of both SUI and urgency symptoms within the first 6 months following RALP. The primary study outcomes included ICIQ‐MLUTS SUI and urgency domain scores and pads per day (PPD).

As part of this programme, patients are also offered directed in‐person PFMT by an FPMRS‐trained specialist (DER). Patients declining in‐person PFMT undergo a standard post‐operative rehabilitation pathway, with pelvic floor exercises directed by the primary oncologic surgeon. Assessment of outcomes related to in‐person versus unsupervised PFMT is the focus of separate research study.

Inclusion criterion was a minimum follow‐up of 6 months.

### 2.1 Statistical analysis

Statistical analysis was performed using SAS version 9.4 (SAS Institute Inc., Cary, NC, USA), and *P*‐values less than 0.05 were considered statistically significant. Data are presented as mean (standard deviation) or *n*(%), as appropriate.

The present analysis focused on SUI (ICIQ‐MLUTS SUI domain score and daily pad use) and urgency (ICIQ‐MLUTS urgency domain score) outcomes following RALP. Accordingly, multivariable linear regression was performed to identify variables associated with SUI and urgency outcomes at 6 months following RALP. The variables of interest included body mass index (BMI), hypertension (HTN), pre‐operative BPH medication use (alpha‐blockers and/or 5‐alpha reductase inhibitors), smoking status, age, pre‐operative prostate specific antigen (PSA), adjuvant external beam radiation therapy (XRT), prostate size, estimated blood loss (EBL), Retzius‐sparing technique, bilateral nerve sparing (BNS), unilateral nerve sparing (UNS) and lymph node dissection (LND). The model was adjusted for the presence of pelvic floor muscle therapy and surgeon. Each variable of interest was run in a separate model with pelvic floor muscle therapy and surgeon to prevent overfitting.

## 3 RESULTS

A total of 40 patients were included in the analysis, undergoing RALP between 2018 and 2021. No patients were excluded for missing data. Patients underwent robotic radical prostatectomy by three different experienced surgeons using techniques including anterior approach, anterior approach with Hood technique reconstruction and Retzius‐sparing approach. Average catheter time was 7–10 days for all patients. Table 1 provides a detailed summary of patient demographics and surgical characteristics.

**TABLE 1:** Patient characteristics.

Patient/disease factors	*n* = 40
Age (years)	62.8 (7.52)
BMI (kg/m^2^)	28.66 (4.22)
Smoking	
Current	5 (12.5%)
Former	11 (27.5%)
Never	23 (57.5%)
Hypertension	19 (47.5%)
Preop BPH med use	8 (20%)
Prostate size (g)	57.2 (33)
PSA (ng/mL)	8.3 (4.5)
Adjuvant XRT	5 (12.5%)

Surgical factors	
EBL (mL)	201 (158)
Nerve sparing	
Bilateral	31 (77.5%)
Unilateral	3 (7.5%)
None	6 (15%)
Retzius‐sparing	8 (20%)
Lymph node dissection	35 (88%)
Post‐operative ISUP grade group	
0	1 (2.5%)
1	7 (17.5%)
2	13 (32.5%)
*3*	13 (32.5%)
*4*	5 (12.5%)
*5*	1 (2.5%)

*Note*: Data presented as mean (SD) or *n* (%) as appropriate.

Abbreviations: BMI, body mass index; BPH, benign prostatic hyperplasia; EBL, estimated blood loss; ISUP, International Society of Urological Pathology; med, medication; PSA, prostate specific antigen; XRT, adjuvant external beam radiation therapy.

The mean patient age and BMI were 62.8 (7.52) and 28.66 (4.22), respectively.

Questionnaire outcomes are detailed in Table 2. In summary, a significant deterioration was seen in SUI and urgency domain scores from baseline to 3 months postoperatively with a significant increase in PPD used. From 3 to 6 months, there was improvement in all outcomes; however, scores remained significantly higher at 6 months compared with baseline.

**TABLE 2:** Patient‐reported urinary outcomes.

	Baseline	3 months	6 months
Outcomes			
ICIQ‐MLUTS SUI domain score	0.075 (0.27)	1.55 (1.2)^a^	1.28 (1.4)^b^
ICIQ‐MLUTS Urgency domain score	0.7 (0.91)	1.25 (1.0)^a^	0.9 (0.78)
Pads per day (*n*)	0.03 (0.16)	1.65 (2.1)^a^	0.9 (1.2)^b^

*Note*: Data presented as mean (SD).

^a^ Significantly differed from baseline to 3 months.

^b^ Significantly differed from baseline to 6 months.

### 3.1 Multivariable analysis

Table 3 shows the multivariable analysis outcomes at 3 months, and Table 4 shows the multivariable analysis outcomes at 6 months.

**TABLE 3:** Predictors for urinary symptom outcomes following ralp (3 months).

	ICIQ‐MLUTS SUI domain score		ICIQ‐MLUTS Urgency domain score		Pads per day (*n*)	
Risk factor	*β* coef	*p*‐value	*β* coef	*p*‐value	*β* coef	*p*‐value
Patient/disease factors						
Age	0.020	0.43	0.0006	0.98	0.053	0.23
BMI (kg/m^2^)	0.091	**0.030**	0.067	0.057	0.21	**0.0028**
Smoking	0.26	0.62	−0.70	0.11	−0.13	0.89
Hypertension	0.66	0.068	0.34	0.27	0.75	0.25
Preop BPH med use	−0.57	0.23	−0.96	**0.0096**	−1.9	**0.015**
Prostate size (g)	0.0023	0.68	−0.0079	0.081	−0.0039	0.69
PSA (ng/mL)	−0.025	0.55	−0.065	0.054	−0.087	0.023
Adjuvant XRT	−0.24	0.66	−0.24	0.60	−0.86	0.37
Surgical factors						
EBL (mL)	0.0013	0.36	0.0	0.99	−0.0010	0.68
Nerve sparing (bilat)	−1.1	0.059	0.0027	0.10	−0.63	0.56
Nerve sparing (unilat)	−1.04	0.18	−0.60	0.38	−0.037	0.98
Retzius‐sparing	−0.34	0.51	−0.28	0.53	−0.21	0.82
Lymph n. dissection	0.14	0.80	−0.22	0.65	1.5	0.12

*Note*: Multivariable linear regression for urinary symptom outcomes (ICIQ‐MLUTS SUI domain score, ICIQ‐MLUTS Urgency domain score and pads per day). Coefficients for each risk factor are controlled for surgeon and pelvic floor muscle therapy. *P*‐values <0.05 are bolded.

Abbreviations: bilat, bilateral; BMI, body mass index; BPH, benign prostatic hyperplasia; EBL, estimated blood loss; lymph n., lymph node; med, medication; PSA, prostate specific antigen; unilat, unilateral; XRT, adjuvant external beam radiation therapy.

**TABLE 4:** Predictors for urinary symptom outcomes following RALP (6 months).

	ICIQ‐MLUTS SUI domain score		ICIQ‐MLUTS Urgency domain score		Pads per day (*n*)	
Risk factor	*β* coef	*p*‐value	*β* coef	*p*‐value	*β* coef	*p*‐value
Patient/disease factors						
Age	0.031	0.26	0.0041	0.80	0.022	0.36
BMI (kg/m^2^)	0.10	**0.026**	0.021	0.46	0.091	**0.022**
Smoking	0.057	0.92	0.026	0.94	0.071	0.90
Hypertension	1.10	**0.0025**	0.16	0.51	0.53	0.13
Preop BPH med use	−0.21	0.69	−0.38	0.21	−0.96	**0.027**
Prostate size (g)	0.0040	0.51	−0.0015	0.68	−0.0040	0.45
PSA (ng/mL)	−0.068	0.13	−0.018	0.51	−0.072	0.060
Adjuvant XRT	0.34	0.57	0.47	0.17	−0.55	0.29
Surgical factors						
EBL (mL)	0.0032	**0.026**	0.0	0.97	−0.00010	0.92
Nerve sparing (bilat)	−1.37	**0.027**	−0.28	0.47	−0.65	0.25
Nerve sparing (unilat)	−1.37	0.099	−0.40	0.44	−1.11	0.15
Retzius‐sparing	−0.88	0.11	−0.23	0.48	−0.014	0.98
Lymph n. dissection	0.63	0.30	−0.45	0.21	0.81	0.13

*Note*: Multivariable linear regression for urinary symptom outcomes (ICIQ‐MLUTS SUI domain score, ICIQ‐MLUTS Urgency domain score and pads per day). Coefficients for each risk factor are controlled for surgeon and pelvic floor muscle therapy. *P*‐values <0.05 are bolded.

Abbreviations: bilat, bilateral; BMI, body mass index; BPH, benign prostatic hyperplasia; EBL, estimated blood loss; lymph n., lymph node; med, medication; PSA, prostate specific antigen; unilat, unilateral; XRT, adjuvant external beam radiation therapy.

### 3.2 SUI

BMI was found to be a significant predictor of worse ICIQ‐MLUTS SUI domain scores at 3 months postoperatively (*β* = 0.091, *p* = 0.030). BMI, HTN and EBL were found to be significant predictors of worse ICIQ‐MLUTS SUI domain scores at 6 months postoperatively (BMI, *β* = 0.10, *p* = 0.026; HTN, *β* = 1.10 p = 0.0025; EBL, *β* = 0.0032, *p* = 0.026). In contrast, bilateral nerve sparing was found to predict better 6‐month ICIQ‐MLUTS SUI domain scores (*β* = −1.37, *p* = 0.027).

### 3.3 Urinary urgency

Pre‐operative BPH medication use was found to be a significant factor in improved urgency domain score at 3 months (*β* = −0.96, *p* = 0.0096). No risk factors were found to significantly predict post‐operative urgency outcomes at 6 months.

### 3.4 Pad use

BMI was significantly associated with greater PPD use postoperatively at 3 months (*β* = 0.21, *p* = 0.0028), while pre‐operative BPH medication use was found to be a significant factor in improved PPD use at 3 months (*β* = −1.9, *p* = 0.015). BMI was significantly associated with greater PPD use postoperatively at 6 months (*β* = 0.091, *p* = 0.022), while pre‐operative BPH medication use was found to be a significant factor in improved PPD use at 6 months (*β* = −0.96, *p* = 0.027).

## 4 DISCUSSION

Incontinence after RALP is a common issue with significant patient bother and negative impact on QOL.^1^, ^2^ Despite national guidelines recommending intervention as early as 6 months postoperatively for persistent bothersome SUI, intervention is often delayed, prolonging decreased QOL.^5^, ^6^ In this post hoc analysis of prospectively collected incontinence outcome data in patients undergoing RALP, we aimed to identify risk factors for worse post‐prostatectomy incontinence at 6 months to facilitate early identification of patients who may benefit from early anti‐incontinence intervention.

We identified the patient factor of higher BMI as a predictor of worse SUI at 3 and 6 months postoperatively. Additionally, HTN was identified as predictors of worse SUI at 6 months postoperatively. While others have also reported BMI predictive of worsened incontinence outcomes following prostatectomy,^6^, ^7^ HTN has previously been found as not predictive.^8^ It is likely that BMI and HTN are colinear and that multivariable modelling in larger population may identify one instead of the other as the true predictor. Overall, prior studies of patient‐specific predictors of post‐prostatectomy incontinence are mixed, with some identifying age, prostate size and smoking status all predictive of worse outcomes,^7^, ^9^, ^10^ while others find these not predictive as was the case in our study.^8^

Interestingly, we also found that preoperative BPH medication use was associated with fewer PPD used at 3 and 6 months postoperatively and decreased urgency at 3 months postoperatively. We used this variable as a surrogate for clinically significant preoperative BPH. This finding is surprising as prior study has also identified preoperative LUTS and predictor of worse post‐prostatectomy incontinence outcomes.^7^ There is little data however on preoperative BPH and BPH medication use in relation to post‐prostatectomy incontinence.^10^ We did not collect information regarding specific BPH medications used (i.e. alpha‐blockers versus 5‐alpha reductase inhibitors); however, one possible explanation for these findings is that the androgen ablative effect of 5‐alpha reductase inhibitors could theoretically favourably affect surgery resulting in better incontinence outcomes.

Our study also identified surgical factors of bilateral nerve sparing and lower EBL as protective against post‐operative SUI at 6 months. These are not surprising. Neural factors have a known essential role in post‐prostatectomy incontinence as denervation of the sphincter disrupts continence,^11^ and the AUA Guideline on Incontinence after Prostate Treatment recognise bilateral nerve‐sparing approach to prostatectomy as only protective surgical manoeuvre linked with improved post‐operative continence.^6^ Additionally, others have also found higher EBL associated with worse SUI outcomes.^12^ EBL could be a marker of increased surgical difficulty, lead to decreased visibility and more injury to neurovascular bundles and/or sphincter.^13^

Although not stated in the AUA guidelines for incontinence after Prostate Treatment, Retzius‐sparing is a surgical technique that has been shown in the literature to reduce post‐RALP SUI.^14^ Umari et al. demonstrated an immediate improvement of incontinence at time of catheter removal; however, there was no difference in ICIQ MLUTS stress incontinence prevalence at 1 month between the Retzius‐sparing RALP and non‐sparing RALP groups.^15^ Liao et al. also demonstrated improved immediate continence, but at 6 months, there was no difference between Retzius‐sparing RALP and RALP groups.^16^ Our analysis demonstrated that there was no significant improvement in SUI outcomes with Retzius‐sparing RALP at 6‐month follow‐up.

Retzius‐sparing approach was not predictive of SUI outcomes at 6 months in this study, which is consistent with prior study. Although this approach has been associated with earlier return to continence on the order of weeks to months,^14^, ^15^ no differences have been found at 6 months compared with standard non‐Retzius‐sparing approaches.^16^

Notably, there were no predictors in our study that were associated with urinary urgency. Overall, urgency is understudied in post‐RALP patients although it is known to be common. It has been shown that urgency occurs in 19%–48% patients post‐prostatectomy,^17^, ^18^ These symptoms can be burdensome, and intervention could benefit QOL. Further study is necessary to investigate this common adverse outcome after prostatectomy.

Our findings can be utilised to guide post‐prostatectomy follow‐up protocols. Patients identified as higher risk for worsened SUI at 6 months, such as obese hypertensive patients who underwent non‐nerve sparing RALP with high EBL, may be followed more closely postoperatively by an FPMRS or GURS specialist. This would allow for comprehensive and improved evaluation and possibly earlier surgical intervention, if indicated. Historically, surgical intervention for SUI was delayed until 12 months post‐RALP; however, recent literature shows that surgical intervention can be initiated at 6 months for persistent bothersome symptoms; as only a small number of patients will improve their continence after 6 months,^6^, ^19^ Nelson et al. demonstrated that the median time for surgical intervention for post‐RALP SUI is 23.5 months.^5^

The study is limited by its retrospective nature and a smaller sample size. The sample size is a function of the programme feasibility. Given the significant time requirements required of a single FPMRS specialist needed to conduct in‐person visits numerous times throughout the post‐operative year, it was not possible to enrol all patients undergoing RALP. That said, we are aware of no other study assessing outcomes in this fashion. Indeed, oncologic surgeons had no access to study data until the analysis phase. In addition, in an effort to account for smaller sample size, our multivariable modelling was limited to considering candidate predictors in isolation while controlling for surgeon and PFMT to avoid over‐fitting. The present cohort is also heterogeneous and, based on the programme participation methodology, subject to bias. That said, we believe that this quality is more consistent with the outcomes seen in community practice and, combined with the strength of validated and longitudinal data, allows urologists further insight into functional outcomes over time that may be used to counsel patients. Additional assessment is ongoing to evaluate other clinical questions including potential functional outcomes differences between in‐person PFMT and standard unsupervised pelvic floor exercises, as well as urgency and SUI outcomes over time.

## 5 CONCLUSION

Increased BMI, EBL and the presence of HTN are associated with worsened SUI outcomes following RALP, whereas bilateral nerve sparing technique and preoperative BPH medication are associated with improved SUI outcomes. These data may inform patient counselling and guide post‐operative follow‐up, as patients with elevated BMI, HTN or higher intraoperative EBL may benefit from closer surveillance and earlier anti‐incontinence intervention.

## AUTHOR CONTRIBUTIONS

All individuals who contributed to the creation of the manuscript have been included in the author section.

## DISCLOSURE OF INTEREST

There are no conflicts of interests among all of the authors.

## References

[1] Stanford JL , Feng Z , Hamilton AS , Gilliland FD , Stephenson RA , Eley JW , et al. Urinary and sexual function after radical prostatectomy for clinically localized prostate cancer: the prostate cancer outcomes study. Jama. 2000;283(3):354–360. 10.1001/jama.283.3.354 10647798

[2] Bauer RM , Gozzi C , Hübner W , Nitti VW , Novara G , Peterson A , et al. Contemporary management of postprostatectomy incontinence. Eur Urol. 2011;59(6):985–996. 10.1016/j.eururo.2011.03.020 21458914

[3] Scott KM , Gosai E , Bradley MH , Walton S , Hynan LS , Lemack G , et al. Individualized pelvic physical therapy for the treatment of post‐prostatectomy stress urinary incontinence and pelvic pain. Int Urol Nephrol. 2020;52(4):655–659. 10.1007/s11255-019-02343-7 31807975

[4] Sullivan JF , Stassen PN , Moran D , Bolton EM , Smyth LG , Browne CM , et al. The transobturator suburethral sling: a safe and effective option for all degrees of post prostatectomy urinary incontinence. Can J Urol. 2018;25(2):9268–9272. 29680005

[5] Nelson M , Dornbier R , Kirshenbaum E , Eguia E , Sweigert P , Baker M , et al. Use of surgery for post‐prostatectomy incontinence. J Urol. 2020;203(4):786–791. 10.1097/JU.0000000000000618 31642741

[6] Sandhu JS , Breyer B , Comiter C , Eastham JA , Gomez C , Kirages DJ , et al. Incontinence after prostate treatment: AUA/SUFU guideline. J Urol. 2019;202(2):369–378. 10.1097/JU.0000000000000314 31059663

[7] Ficarra V , Novara G , Rosen RC , Artibani W , Carroll PR , Costello A , et al. Systematic review and meta‐analysis of studies reporting urinary continence recovery after robot‐assisted radical prostatectomy. Eur Urol. 2012;62(3):405–417. 10.1016/j.eururo.2012.05.045 22749852

[8] Wille S , Heidenreich A , von Knobloch R , Hofmann R , Engelmann U . Impact of comorbidities on post‐prostatectomy incontinence. Urol Int. 2006;76(3):223–226. 10.1159/000091623 16601383

[9] Novara G , Ficarra V , D'elia C , Secco S , Cioffi A , Cavalleri S , et al. Evaluating urinary continence and preoperative predictors of urinary continence after robot assisted laparoscopic radical prostatectomy. J Urol. 2010;184(3):1028–1033. 10.1016/j.juro.2010.04.069 20643426

[10] Rajih E , Meskawi M , Alenizi AM , Zorn KC , Alnazari M , Zanaty M , et al. Perioperative predictors for post‐prostatectomy urinary incontinence in prostate cancer patients following robotic‐assisted radical prostatectomy: long‐term results of a Canadian prospective cohort. Can Urol Assoc J. 2018;13(5):E125–E131. 10.5489/cuaj.5356 30332593 PMC6520054

[11] Bessede T , Sooriakumaran P , Takenaka A , Tewari A . Neural supply of the male urethral sphincter: comprehensive anatomical review and implications for continence recovery after radical prostatectomy. World J Urol. 2017;35(4):549–565. 10.1007/s00345-016-1901-8 27484205

[12] Lazarovich A , Abu‐Ghanem Y , Rosenzweig B , Dotan ZA , Zilberman DE . Factors predicting full urinary continence following robot‐assisted laparoscopic radical prostatectomy (RALP). Harefuah. 2021;160(9):594–597. 34482672

[13] Preisser F , Pompe RS , Salomon G , Rosenbaum C , Graefen M , Huland H , et al. Impact of the estimated blood loss during radical prostatectomy on functional outcomes. Urol Oncol. 2019;37(5):298.e11–298.e17. 10.1016/j.urolonc.2019.01.006 30857988

[14] Davis M , Egan J , Marhamati S , Galfano A , Kowalczyk KJ . Retzius‐sparing robot‐assisted robotic prostatectomy: past, present, and future. Urol Clin North am. 2021;48(1):11–23. 10.1016/j.ucl.2020.09.012 33218585

[15] Umari P , Eden C , Cahill D , Rizzo M , Eden D , Sooriakumaran P . Retzius‐sparing versus standard robot‐assisted radical prostatectomy: a comparative prospective study of nearly 500 patients. J Urol. 2021;205(3):780–790. 10.1097/JU.0000000000001435 33086025

[16] Liao P‐C , Hung S‐C , Hu J‐C , Chiu K‐Y . Retzius‐sparing robotic‐assisted radical prostatectomy facilitates early continence regardless of neurovascular bundle sparing. Anticancer Res. 2020;40(7):4075–4080. 10.21873/anticanres.14405 32620655

[17] Gomha MA , Boone TB . Voiding patterns in patients with post‐prostatectomy incontinence: urodynamic and demographic analysis. J Urol. 2003;169(5):1766–1769. 10.1097/01.ju.0000059700.21764.83 12686829

[18] Hosier GW , Tennankore KK , Himmelman JG , Gajewski J , Cox AR . Overactive bladder and storage lower urinary tract symptoms following radical prostatectomy. Urology. 2016;94:193–197. 10.1016/j.urology.2016.05.007 27181241

[19] De Carlo F , Celestino F , Verri C , Masedu F , Liberati E , Di Stasi SM . Retropubic, laparoscopic, and robot‐assisted radical prostatectomy: surgical, oncological, and functional outcomes: a systematic review. Urol Int. 2014;93(4):373–383. 10.1159/000366008 25277444

